# Gastric Cancer: Pathobiology and Therapeutics

**DOI:** 10.1002/mco2.70772

**Published:** 2026-05-23

**Authors:** Ruixian Yu, Miao Zhang, Yan Meng, Chunxiao Zhu, Weihong Zhang, Hui Zhang, Zhifa Cao, Meihang Du, Zhangting Zhao, Junping Bai, Yi Han, Yang Tang, Wei Kang, Ka Fai To, Shi Jiao, Liwei An, Zhaocai Zhou

**Affiliations:** ^1^ State Key Laboratory of Genetics and Development of Complex Phenotypes, School of Life Sciences Department of General Surgery, QingPu Branch of Zhongshan Hospital, Fudan University Shanghai China; ^2^ Department of Stomatology, Shanghai Tenth People's Hospital, Department of Biochemistry and Molecular Biology Tongji University Cancer Center, School of Medicine, Tongji University Shanghai China; ^3^ Department of Anatomical and Cellular Pathology, State Key Laboratory of Translational Oncology, State Key Laboratory of Digestive Disease Prince of Wales Hospital, The Chinese University of Hong Kong Hong Kong China; ^4^ Tianfu Jincheng Laboratory Chengdu China

**Keywords:** gastric cancer, immunotherapy, molecular classification, neural regulation, targeted therapy, tumor microenvironment

## Abstract

Gastric cancer (GC) remains a formidable global health challenge, characterized by pronounced molecular heterogeneity, late‐stage diagnosis, and limited durable responses to existing therapies. This review synthesizes recent advances in GC research through an integrated, multidisciplinary lens, spanning tumor biology, microenvironmental dynamics, and therapeutic innovation. We first consolidate updated histopathological and molecular classification systems, highlighting oncogenic programs that underpin GC development, including Hippo‐YAP signaling and emerging neural–stem cell interactions. We then examine the immunosuppressive tumor microenvironment, emphasizing the dynamic crosstalk among tumor‐associated macrophages, regulatory T cells, tertiary lymphoid structures, and cancer‐associated fibroblasts that collectively drive metastatic dissemination and therapeutic resistance. Emerging biomarker‐guided strategies, including CLDN18.2‐targeted therapies, dual immune checkpoint blockade, and engineered cellular therapies, are critically discussed alongside rational combination approaches designed to overcome resistance. Beyond canonical paradigms, we highlight transformative frontiers, such as cancer neuroscience, microbiome‐driven immune modulation, and spatially resolved multiomics technologies, that enable high‐resolution mapping of cellular interactions. Finally, we critically assess translational barriers, including organ‐specific metastatic tropism and resistance evolution, and propose that the convergence of deep molecular profiling, neural‐immune modulation, and AI‐enabled computational oncology will be central to advancing precision medicine for GC. This integrated framework aims to accelerate the development of mechanism‐based combination therapies.

## Graphical abstract

Gastric cancer is a multistep process driven by the interplay of microbial virulence, host epithelial injury, and evolving molecular reprogramming. Initiation involves pathogen‐induced epigenetic alterations and the early subversion of key oncogenic signaling networks, facilitating the transition from chronic injury to metaplasia and malignant transformation. This progression is reinforced by neuroimmune crosstalk that further promotes tumor development. As the tumor evolves, it establishes a complex immunomodulatory microenvironment characterized by an extensive cytokine network that governs immune dynamics and sustains therapeutic resistance. Together, these initiation and adaptation programs define the trajectory from healthy mucosa to treatment‐refractory gastric cancer.

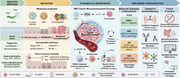

## 1 Introduction

Gastric cancer (GC) remains a major global health burden, ranking as the fifth most common malignancy and the third leading cause of cancer‐related mortality worldwide [1], accounting for over 700,000 deaths annually [2]. Despite significant advances in understanding environmental risk factors, refining endoscopic detection, and evolving surgical techniques, the clinical reality remains sobering: a substantial proportion of patients are diagnosed at advanced stages, where metastatic spread and limited treatment options translate into poor long‐term survival. Moreover, early detection is often hindered by nonspecific clinical symptoms, uneven implementation of screening programs, and the biological heterogeneity of precancerous lesions, collectively contributing to delayed diagnosis and suboptimal prognosis.

Over the past decade, GC research has undergone a paradigm shift, namely, from a predominantly histopathology‐centered view to an integrative framework that encompasses molecular stratification, tumor microenvironment (TME) ecology, and dynamic tumor evolution. Large‐scale genomic and multiomics studies have proposed molecular classification systems that capture distinct etiologies, immune states, and therapeutic vulnerabilities, while simultaneously revealing extensive intra‐ and intertumoral heterogeneity [3, 4, 5]. Concurrently, emerging evidence highlights that GC progression and treatment resistance are shaped not only by tumor‐intrinsic alterations but also by evolving interactions with immune and stromal compartments, metabolic adaptation, neural crosstalk, and organ‐specific metastatic niches [4, 6, 7, 8, 9]. Although these advances have facilitated the emergence of targeted therapies and immunotherapy, durable clinical benefits remain limited to selected patient subsets, highlighting ongoing challenges in translating mechanistic insights into broadly effective clinical interventions.

Several comprehensive reviews have addressed individual aspects of GC biology and treatment, including molecular subtypes, immune regulation, and therapeutic strategies [10, 11, 12, 13, 14]. However, a synthesis that explicitly links molecular classification to multidimensional pathogenesis, TME dynamics and the evolving landscape of therapy resistance is still needed. However, these studies lack a holistic perspective that integrates molecular subtyping with the complex, multidimensional mechanisms underlying tumor progression and treatment resistance. This is particularly true for emerging regulatory pathways that have garnered increasing attention in recent years—such as neuro–tumor interactions and microbiome‐mediated regulatory mechanisms. Although growing functional evidence supports their critical roles, they have not yet been fully incorporated into existing research frameworks. Therefore, there is an urgent need for systematic integrative analyses to bridge the gaps between different research dimensions, refine current theoretical models, and provide directional guidance for future translational research.

In this review, we aim to provide a comprehensive and clinically relevant framework that integrates recent advances in GC research across multiple levels. We begin by summarizing the epidemiology and molecular classification of GC, establishing the foundation for understanding disease heterogeneity. We then discuss key mechanisms driving tumor initiation and progression, with a particular focus on TME dynamics, including immune regulation, stromal interactions, metabolic adaptation, and emerging neural and microbial influences. Subsequently, we examine current therapeutic strategies, including targeted therapy, immunotherapy, and combination approaches, with an emphasis on mechanisms of treatment resistance and response heterogeneity. Finally, we highlight unresolved challenges and future directions, proposing potential strategies for biomarker‐driven precision medicine and rational therapeutic combinations. Through this structured overview, we aim to provide a coherent roadmap linking fundamental biology to clinical application in GC.

## 2 Epidemiology and Classification

GC is a highly heterogeneity cancer in terms of its epidemiology across the world, pathological morphology, and molecular signatures, which helps understand the common features of a certain subtype and contributes to precision medicine.

### 2.1 Epidemiological Landscape

GC demonstrates striking geographical disparities, with highest incidence in Eastern Asia versus Western countries [15]. While Western nations show declining overall rates but rising proximal tumors [16, 17], Eastern populations demonstrate lower frequencies of signet‐ring histology and proximal involvement [18, 19, 20]. Male predominance and rising proximal tumors reflect differential exposure to risk factors including *Helicobacter pylori* infection, lifestyle habits (smoking, alcohol, and preserved foods), and genetic susceptibility [21, 22].

**Key modifiable risk factors for GC** include ***H. pylori*** infection, smoking, and diets high in nitrates/nitrites [23]. *H. pylori*, a Gram‐negative bacterium identified in 1983 as the primary cause of peptic ulcers [24, 25], represents a dominant environmental risk for distal GC. While infecting 50% of humans globally [26], less than 5% of carriers develop cancer due to strain variations, host genetics, infection timing, and environmental cofactors [27]. While cardia cancers occasionally associate with *H. pylori*‐induced gastric atrophy, they typically show no infection correlation or even negative association in certain populations [28, 29].

**Epstein–Barr virus (EBV)**, while classically associated with lymphoproliferative malignancies, infects gastric epithelial cells to drive lymphoepithelioma such as carcinoma. This subtype is characterized by stromal lymphocyte infiltration (predominantly CD8^+^ T cells), high lymph node metastasis rate [30] and PD‐L1 overexpression, suggesting immunomodulatory approaches may be particularly effective in this subtype.

### 2.2 From Traditional Classification to Molecular Subtyping

#### 2.2.1 Grading and Staging

**FIGURE 1:**
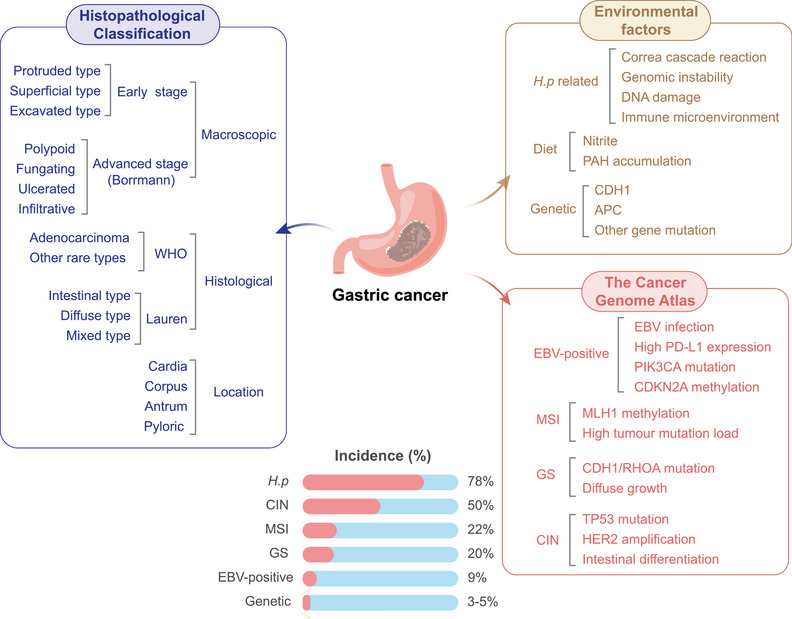
Gastric cancer (GC) epidemiology and classification. GC is a biologically and clinically heterogeneous disease shaped by the interplay of environmental exposure, host susceptibility, and molecular evolution. Its classification has traditionally relied on gross morphology, histology, and anatomical location, whereas contemporary molecular frameworks further subdivide tumors into genomically distinct groups with different biological and therapeutic implications. Together, these clinicopathological and molecular perspectives provide a complementary framework for understanding disease origins, population patterns, and the diversity of GC behavior.

GC is grossly classified as early or advanced, and histologically graded as highly (G1), moderately (G2), or poorly differentiated/undifferentiated (G3). Staging follows the AJCC/UICC eighth edition system, encompassing clinical (cTNM), pathological (pTNM), and postneoadjuvant therapy pathological (ypTNM) staging. cTNM assesses tumor size (T), nodal status (N), and distant metastasis (M) via endoscopy and imaging [31, 32, 33]. Endoscopic ultrasonography (EUS) is particularly useful for detecting early‐stage tumors (AJCC Stage I) amenable to endoscopic resection or surgery. However, most patients present at Stages II–IV, where EUS and conventional imaging have limited accuracy for detecting lymph node metastases. While standard chest, abdominal, or pelvic CT generally sufficient for staging, FDG‐PET/CT may be considered for specific indications, such as further evaluation of indeterminate lesions [31, 33] (Figure 1).

#### 2.2.2 Histological Classification

**Histological classification of GC** is primarily based on the WHO and Lauren systems. The fifth edition of the WHO classification (2019) categorizes GCs into adenocarcinoma (> 90%) and rare histological types. Adenocarcinoma is further divided into papillary, tubular, mucinous, and low‐adhesion carcinomas (including signet‐ring cell carcinoma), with newly added rare subtypes such as hepatoid and medullary carcinomas. This classification also emphasizes integrated diagnosis based on molecular features like microsatellite instability.

**Lauren's system** classifies gastric adenocarcinoma into three subtypes based on histopathological features [34]. Intestinal‐type GCs show glandular formations with goblet cells and moderate differentiation. Diffuse‐type GCs consist of poorly cohesive cells lacking glandular structure, driven by CDH1 inactivation (E‐cadherin loss) [35] and enriched for RHOA mutations (15%–25%) and PSCA polymorphisms [36, 37, 38]. Mixed‐type tumors exhibit features of both. Intestinal‐type GCs are associated with chromosomal instability (CIN), APC/TP53 mutations, and HER2 amplification. Transcriptomic profiling has further defined therapeutically relevant subtypes [39] (Figure 1).

#### 2.2.3 The TCGA Molecular Subtyping Framework

The integration of multiomics profiling has refined GC classification beyond histology. The Cancer Genome Atlas (TCGA) defines four molecular subtypes with distinct pathogenesis and therapeutic implications (Figure 1) [40, 41], providing a biological rationale for precision oncology.

##### 2.2.3.1 EBV‐Positive (8%–10%)

This subtype is characterized by extreme CpG island hypermethylation (CIMP phenotype), frequent PIK3CA mutations (around 80%), ARID1A truncations (73%), and PD‐L1/PD‐L2 overexpression, which is often driven by JAK2 amplification in about 40% of cases. This subtype has the most favorable prognosis, with a 5‐year overall survival of 65%. These patients also respond well to PD‐1 inhibitors, with objective response rates ranging from 45% to 60%.

##### 2.2.3.2 Microsatellite Unstable (MSI, 15%–22%)

This subtype is defined by MLH1 silencing (70%–80% of cases), ARID1A mutations (83%), and high tumor mutational burden due to mismatch repair deficiency. This subtype is more common in Asian populations (22%) than in Western ones (15%) [42]. First‐line pembrolizumab is approved for this subtype, although JAK1/2 mutations seen in about a quarter of patients may contribute to therapy resistance.

##### 2.2.3.3 Genomically Stable (GS, 20%–25%)

This subtype largely corresponds to diffuse‐type histology, which often contains signet‐ring cells. Key genetic alterations include CDH1 inactivation (37%), RHOA mutations (15%, mainly G17V hotspot), and CLDN18‐ARHGAP fusions (30%). On the treatment side, CLDN18.2 is an emerging target. Zolbetuximab targeting CLDN18.2 has already succeeded in Phase III trials. Rho‐kinase inhibitors are also being explored, though still at the preclinical stage.

##### 2.2.3.4 Chromosomally Unstable (CIN, 50%)

This subtype is marked by widespread aneuploidy and association with intestinal‐type histology. TP53 mutations are seen in 71% of cases, along with amplifications of receptor tyrosine kinases such as HER2 (24%), EGFR (15%), and FGFR2 (9%) [43, 44].

#### 2.2.4 Limitations of Current Classification Systems and the Need for Integration

Despite the biological insights provided by molecular classification, several limitations hinder its direct clinical application. First, the cost and turnaround time of comprehensive genomic profiling remain barriers to routine implementation, particularly in regions with high GC incidence but limited resources. Second, most molecular classifications are derived from primary tumors, yet metastatic lesions, closely related to patient outcomes, often exhibit divergent genomic features, limiting the utility of primary tumor‐based stratification in advanced disease. Third, the unique epidemiological and genetic landscape of GC in East Asian populations, shaped by distinct dietary, lifestyle, and host genetic factors, may not be fully captured by classification systems derived predominantly from Western cohorts. Fourth, the dynamic evolution of tumor clones under therapeutic pressure necessitates longitudinal profiling, which is rarely incorporated into static classification frameworks.

These limitations underscore the need for an integrated model that combines TNM staging, histological characteristics, key molecular biomarkers (e.g., HER2, MSI, PD‐L1, CLDN18.2), and emerging features such as TME immune contexture and neural infiltration. Such a multidimensional approach will be essential for accurate prognosis prediction and therapeutic guidance in the era of precision oncology.

## 3 Multidimensional Pathogenesis

GC development is a multistep and complex process, which progresses from mucosal injury to intestinal metaplasia and finally early onset of tumorigenesis. This section reviews established molecular mechanisms driving the stepwise pathogenesis of GC.

### 3.1 Gastric Mucosal Injury: The Initiating Step

#### 3.1.1 Core Concept

Gastric mucosal injury serves as the common entry point for gastric carcinogenesis. The pattern of injury, including focal versus diffuse, determines subsequent repair mechanisms and metaplastic outcomes.

Gastric mucosal injury manifests in two distinct forms that shape downstream pathological tranectories [45]. Focal injury, typically caused by toxins, bile reflux, or pathogens, is characterized by preserved cellular differentiation patterns and rapid healing through neighboring cell proliferation and migration [46, 47, 48]. In contrast, diffuse injury involves widespread epithelial damage that triggers altered cellular differentiation, leading to spasmolytic polypeptide‐expressing metaplasia (SPEM) and intestinal metaplasia [49, 50]. This chronic condition not only causes clinical symptoms including abdominal pain, anemia, ulceration [51], but also significantly elevates cancer risk [52].

**FIGURE 2:**
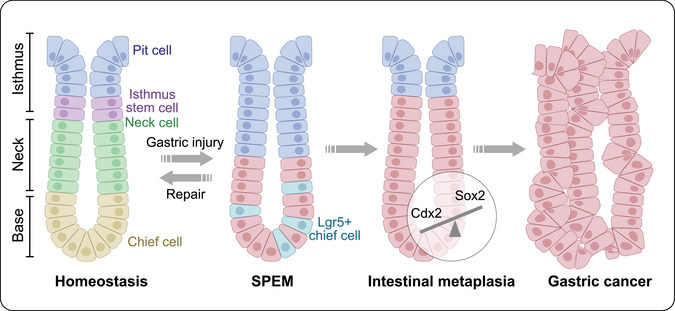
Schematic illustration of the pathological dynamics of gastric tumorigenesis. Gastric tumorigenesis is driven by progressive epithelial disorganization following persistent mucosal injury. Although acute injury may be resolved through tissue repair, chronic injury and inflammation favor metaplastic conversion, lineage reprogramming, and aberrant differentiation, thereby promoting intestinal metaplasia and subsequent malignant transformation.

While early research focused on parietal cell loss as the primary initiator of diffuse injury [48], contemporary studies increasingly emphasize how *H. pylori* orchestrates mucosal damage through multiple pathogenic mechanisms (Figure 2). *H. pylori* evades host immune responses and persists in the gastric mucosa [53, 54]. This chronic colonization, shaped by bacterial virulence factors and host genetics, drives the development of gastric lesion [55]. Infection triggers epithelial cytokine release and macrophages recruit [56]. Subsequent epithelial damage allows bacterial penetration into the lamina propria and lymph nodes, where *H. pylori* activates macrophages to release inflammatory mediators that exacerbate tissue injury [57]. Key virulence factors include CagA, VacA, and outer membrane adhesins (e.g., BabA, SabA), each promoting carcinogenesis through distinct mechanisms [58, 59, 60, 61, 62]. Among these, CagA shows the strongest epidemiological and mechanistic link to GC [59, 60, 61, 62, 63, 64].

Following gastric epithelial injury, particularly parietal cell loss, chief cells undergo transdifferentiation into mucus‐secreting cells, defining SPEM [65, 66]. During chronic inflammation, SPEM progresses to neoplasia [67], establishing chief cells as the primary origin of injury‐induced metaplasia. RUNX3 maintains chief cell differentiation and its dysregulation promotes carcinogenesis [68, 69]. Lgr5^+^ chief cells can function not only as facultative stem cells during epithelial repair, but also as a cellular origin of GC to initiate tumorigenesis upon oncogenic stimulation [70].

Drug‐ and stress‐induced gastric mucosal injury operates through distinct mechanisms. For example, aspirin inhibits cyclooxygenase (COX), reduces cellular ATP, alters sodium transport, increases proton leakage, and disrupts hydrophobic barrier function [71]. Alcohol directly damages epithelial cells via membrane disruption, triggering oxidative stress, and leading to proinflammatory activation [72]. Neurogenic stress is a whole different story. It involves multiple brain regions, including the hypothalamus, brainstem, and limbic system [73]. For instance, restraint water‐immersion stress activates the ventromedial hypothalamic nucleus, exacerbating gastric injury [74, 75].

### 3.2 Gastric Intestinal Metaplasia

Gastric intestinal metaplasia (GIM), which is characterized by the replacement of gastric mucosa with intestinal‐type epithelium, is a pivotal precancerous lesion in gastric carcinogenesis (Figure 2) [76, 77]. This reprogramming involves ectopic expression of intestinal cell lineages [78, 79]. Histologically, GIM is classified as incomplete (mixed), featuring both gastric and intestinal phenotypes, or complete (intestinal), with fully differentiated intestinal epithelium.

Although its etiology is multifactorial, chronic *H. pylori* infection is the primary driver, initiating a cascade from chronic inflammation to atrophic gastritis, GIM, and dysplasia [80]. Dietary carcinogens (e.g., MNNG), bile reflux and microbial dysbiosis may synergistically promote GIM [81]. Clinically, early GIM may regress after *H. pylori* eradication [82], whereas established GIM progresses irreversibly despite pathogen clearance [83, 84]. It may be attributed to an epigenetic “field effect” that locks in the intestinal differentiation program. This distinction is critical for risk stratification and surveillance.

Molecularly, GIM is driven by CDX2 (a master regulator of intestinal differentiation [85]) overexpression, Wnt/β‐catenin activation (inducing stem cell reprogramming) and TFF3 suppression (loss of gastric phenotype). Ectopically expressed CDX factors regulate proliferation, apoptosis, and adhesion, promoting intestinal columnar phenotypes [86]. Mechanistically, CDX2 upregulation coupled with SOX2 downregulation drives intestinal transdifferentiation [87]. CDX1 directly activates SALL4 and KLF5 to convert gastric epithelial cells into intestinal progenitors [88] and is transcriptionally regulated by Wnt/β‐catenin [89]. IL‐6/STAT3 signaling also induces GIM via the STAT3–CDX2 axis [90].

Liang et al. demonstrated that *H. pylori* induces GIM characterized by CDX2 and MUC2 upregulation [91]. This effect is mediated by KAT2‐driven activation of the kynurenine pathway, producing xanthurenic acid that directly induces CDX2 expression. Concurrent sonic hedgehog (Shh) deficiency correlates with metaplastic progression [92, 93]. Thus *H. pylori* simultaneously activates metaplasia‐promoting factors (CDX2) while repressing gastric identity maintainers (Shh), orchestrating intestinal transdifferentiation.

### 3.3 Early Onset of GC

Malignant transformation requires cancer hallmarks including autonomous proliferation apoptosis resistance [94, 95]. This section summarizes key genetic and molecular alterations driving gastric epithelial cell malignant transformation (Figure 2).

#### 3.3.1 Altered Signaling Pathways in GC Initiation

**FIGURE 3:**
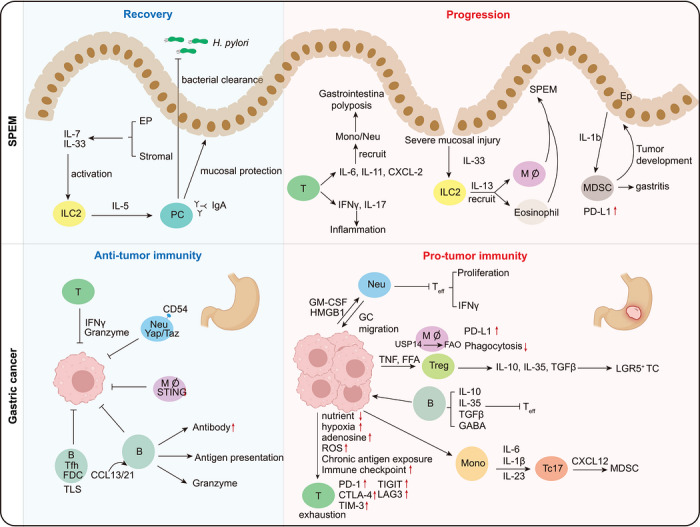
Various components of TME and signaling pathways regulating immune evasion. The gastric microenvironment is dynamically reshaped during the transition from chronic mucosal injury to overt malignancy. In the early setting, immune responses can support epithelial repair, barrier integrity, and microbial control. However, when injury and inflammation persist, these protective circuits are progressively replaced by immunosuppressive and tumor‐promoting programs that facilitate metaplastic progression, epithelial plasticity, and malignant transformation. In established gastric cancer, this imbalance is further reinforced by reciprocal interactions among tumor cells, myeloid and lymphoid populations, stromal components, and soluble mediators, ultimately fostering immune evasion, weakening effective antitumor surveillance, and sustaining tumor growth.

**Acquisition of autonomous proliferation** involves multiple dysregulated pathways: cell cycle [96, 97], Hippo [98, 99], Wnt/β‐Catenin [100], PI3K/AKT [101, 102, 103], MAPK [104], NF‐kB [105, 106], JAK/STAT [107, 108, 109], and p53 [110]. Among these, cell cycle is particularly critical. Cyclins, cyclin‐dependent kinases (CDKs), and CDK inhibitors (CDKIs) govern proliferation and differentiation [111]. In GC, frequent overexpression of cell cycle regulators, such as CDK4 (48%), cyclin D1 (34%), cyclin D2 (30%), and cyclin E (44%), enables sustained proliferative signaling [112]. Notably, CDK4 and cyclin D2 overexpression independently predict tumor progression [113, 114, 115] (Figures 3).

**Hippo‐YAP signaling** has emerged as a central regulator of GC proliferation and stemness. Our group and others have identified dysregulation of key Hippo pathway components in GC, including STRN3 [98], MST4 [98, 99], VGLL4, IRF3 [116], AARS1 [117], and OLFM4 [118]. These alterations drive tumor proliferation in vivo and in vitro, establishing the Hippo pathway as a promising therapeutic target [119]. We recently developed an innovative strategy using a VGLL4‐mimetic “glue” peptide that selectively induces TEAD4‐repressive biomolecular condensation, exerting potent antitumor effects [120]. Furthermore, we uncovered a previously unrecognized role of the STRIPAK complex in DNA double‐strand break repair and proposed cotargeting STRIPAK‐PARP as a synthetic lethality strategy for GC [121]. More recently, we identified alanyl‐tRNA synthetase 1 (AARS1) as a bona fide lactyltransferase that directly catalyzes protein lactylation in response to lactate accumulation, activating YAP‐TEAD signaling to promote GC progression [117], which revealing a direct link between metabolic reprogramming and Hippo pathway activation.

**Dysregulation of antiapoptotic mechanisms** is equally critical in gastric carcinogenesis. The tumor suppressor p53 orchestrates DNA damage responses by inducing cell cycle arrest and apoptosis, thus loss‐of‐function TP53 mutations promote tumorigenesis [122]. p53 alterations include aberrant expression, mutations and loss of heterozygosity, which are prevalent in gastric carcinomas and precursors [123]. TCGA data reveal TP53 mutations in 49% of CIN‐subtype GCs, with missense variants in 24% of MSI and 11% of GS subtypes [124]. In CIN‐type GCs, elevated MDM2 and MDM4 expression enhances p53 ubiquitination and proteasomal destruction [42, 125].

#### 3.3.2 Emerging Histone Modifications: Bridging Metabolism and Epigenetics

**DNA methylation** involves methyl transfer to cytosine 5′ position, forming 5‐methylcytosine [126, 127], catalyzed by DNA methyltransferases (DNMTs) [128]. All three DNMTs are overexpressed in GC and correlate with poor prognosis [129, 130]. Methylation primarily targets promoter CpG islands and hypermethylation induces transcriptional silencing [131, 132]. Interestingly, cancer cells exhibit global hypomethylation coupled with focal promoter hypermethylation [133, 134], which is a key mechanism for tumor suppressor inactivation in human cancers [135, 136, 137, 138, 139, 140, 141]. For example, aberrant methylation of cell cycle regulators drives GC proliferation, such as p16^INK4^, a CDK4/6‐cyclin D1 inhibitor [142, 143], and RASSF1A, a G1 arrest regulator [144, 145, 146]. Notably, infection also plays a role in dysregulation of methylation in GC. *H. pylori* infection hypermethylates several important genes, including CDH1, p16^INK4^, APC, MLH1, COX2, CDKN2A, and GNAS [147, 148, 149, 150, 151] while EBV induces genome‐wide methylation, which targets p14^ARF^, p16^INK4^, and RUNX3 [152, 153, 154].

**Histone modifications**, including methylation, acetylation, phosphorylation, ubiquitination, establish a “histone code” that modulates chromatin architecture and RNA polymerase accessibility, thereby regulating gene expression [155, 156, 157, 158, 159]. Aberrant histone modifications are significantly implicated in gastric carcinogenesis [160].

**Histone methylation** is more complex than DNA methylation because it can happen on different residues and in different forms. For example, arginine can be mono‐ or di‐methylated while mono‐, di‐, or tri‐methylation for lysine [161, 162]. In GC, repressive marks such as H3K9me2 and H3K27me3 silence tumor suppressors like CDH1. The abundance of these marks tends to predict poor prognosis [163]. On the other hand, activating marks like H3K4me3 promote carcinogenesis via SETD1A‐mediated glycolytic gene activation [164]. Apart from lysine methylation, arginine methylation has also been reported in GC development. PRMT5 cooperates with c‐Myc to repress tumor suppressors through H4R3me2 [165].

**Histone acetylation** is also dysregulated in GC, which is added by HATs and removed by HDACs [166]. Some HATs act as tumor suppressors. PCAF/Tip60, for example, are downregulated in GC and TIP60 loss correlates with invasion and metastasis [167, 168]. Conversely, HDAC1/2 tend to be overexpressed in GC, which associates with advanced disease and worse survival [169, 170].

Growing evidence has uncovered metabolite‐mediated novel histone modifications, including lactylation [171], itaconation [172], sialylation [173], aminoacylation [174], and monoaminylation [175]. Among these, protein monoaminylation represents a distinctive biochemical process wherein biogenic monoamines (e.g., serotonin, dopamine, histamine) are covalently conjugated to protein substrates via transglutaminase 2 (TGM2)‐mediated transamidation of glutamine residues [176, 177]. To date, three endogenous monoamine‐derived modifications—serotonylation [178, 179, 180], dopaminylation [181, 182], and histaminylation [183] have been characterized as dynamic regulators of chromatin structure and transcriptional programs. A pivotal advance was the discovery of site‐specific histone monoaminylation (particularly at H3Q5), establishing a direct link between neurotransmitter signaling and epigenetic gene regulation [20]. Given the rich innervation of the stomach and the established role of neurotransmitters in gastric physiology, these modifications may represent an unexplored layer of neural–epithelial crosstalk in gastric carcinogenesis, which is a frontier ripe for investigation.

#### 3.3.3 miRNAs and lncRNAs in GC Initiation

In addition to epigenetic modifications, microRNAs (miRNAs) and long noncoding RNAs (lncRNAs) play key roles in gastric carcinogenesis. miRNAs, approximately 18–25 nt, typically suppress gene expression by binding to the 3′ UTRs of target mRNAs, leading to translational repression or degradation [184]. In GC, tumor‐suppressive miRNAs such as miR‐145, miR‐596, and miR‐31 are frequently downregulated and correlate with better prognosis [185, 186, 187], while oncogenic miRNAs like miR‐421 and miR‐106a are upregulated and promote tumor progression [188, 189]. Notably, tumor‐derived miRNAs in serum remain stable and show promise as noninvasive biomarkers [190]. A panel of five serum miRNAs (miR‐1, miR‐20a, miR‐27a, miR‐34, and miR‐423‐5p) has demonstrated superior diagnostic sensitivity for GC compared to conventional biomarkers [191].

LncRNAs (> 200 nt) regulate gene expression through diverse mechanisms, notably by acting as competitive endogenous RNAs (ceRNAs) that sponge miRNAs and thereby restore target mRNA expression [192, 193]. Multiple lncRNAs are implicated in GC initiation and progression. For example, NEAT1 is upregulated and promote proliferation and metastasis by sponging miR‐1294 and activating AKT1 [194]. MEG3 is downregulated and functions as tumor suppressor by sequestering oncogenic miR‐181 [195]. HNF1A‐AS1 is overexpressed and drives GC progression through upregulation of CDK2, CDK4 and cyclin E1 [97].

#### 3.3.4 Neural Regulation of GC Initiation

The gastrointestinal tract is innervated by both intrinsic enteric and extrinsic autonomic nervous systems [196, 197]. Emerging evidence points to a direct role for neural signaling in GC initiation. Vagotomy suppresses gastric tumorigenesis in multiple animal models, implicating vagus‐derived acetylcholine (ACh) in promoting carcinogenesis [198, 199]. Mechanistically, ACh activates muscarinic receptor M3 on epithelial cells, driving proliferation and inhibiting apoptosis [200, 201]. Conversely, stress‐induced sympathetic neurotransmitters such as norepinephrine and epinephrine promote GC progression by activating β‐adrenergic receptors (ADRB) in the TME. Specifically, ADRB2 signaling enhances proliferation and invasion [202, 203]. Its pharmacological blockade induces cell cycle arrest and apoptosis in vitro, partly through downregulation of oncogenic transcription factors such as NF‐κB, AP‐1, and STAT3.

In addition to autonomic nerves, sensory neurons undergo marked expansion in GC. Mouse models have revealed NGF‐dependent proliferation of CGRP^+^ peptidergic nociceptive nerves within tumors [9]. These sensory neurons form functional circuits with cancer cells. Chemogenetic activation triggers calcium flux in tumor cells and accelerates growth and metastasis, while sensory nerve ablation or CGRP blockade suppresses tumor progression and prolongs survival. Optogenetic stimulation of gastric tumors induces calcium responses in the central jugular nucleus and CGRP release, revealing a bidirectional cancer–sensory neuron axis. This peptidergic pathway, distinct from synaptic interactions observed in CNS tumors, highlights sensory innervation as a potential organ‐specific therapeutic target in GC. Collectively, these findings illustrate how GC co‐opts both autonomic neurotransmitters and sensory neuropeptides through dedicated neural circuits to drive tumorigenesis.

## 4 Dynamic Tumor Microenvironment

The gastric TME comprises a dynamic ecosystem of immune cells, stromal components, and signaling molecules that collectively influence cancer initiation, progression, and therapeutic response. This section examines key innate and adaptive immune populations shaping gastric carcinogenesis.

### 4.1 Innate Immunity

#### 4.1.1 ILC2s Regulate SPEM in the Stomach

Innate lymphoid cells (ILCs) maintain gastric mucosa homeostasis and immune surveillance. Among ILC subsets, type 2 innate lymphoid cells (ILC2s) are enriched in the stomach and serve a dual function in host defense and tissue repair. During *H. pylori* infection, epithelial‐derived IL‐7 and IL‐33 activate ILC2s, which enhance IgA production by plasma cells via the IL‐7–IL‐7R axis to promote bacterial clearance [204].

Beyond antimicrobial defense, ILC2s critically drive SPEM following gastric injury. After acute epithelial damage, ILC2s accumulate in the mucosa and secrete IL‐13 in an IL‐33‐dependent manner. IL‐13 orchestrates recruitment of reparative cell populations, including alternatively activated macrophages and eosinophils, promoting metaplastic remodeling. Genetic or pharmacologic ILC2 depletion suppresses tuft cell expansion and SPEM formation after L635‐induced injury [205], establishing ILC2‐driven type 2 immunity as essential for metaplasia development.

Single‐cell and spatial transcriptomics have revealed substantial cellular heterogeneity within SPEM lesions, challenging the concept of SPEM as a uniform epithelial state [206]. ILC2‐derived IL‐13 functions as a niche signal shaping epithelial cell fate decisions. The immune landscape accompanying SPEM is highly dynamic: ILC2 activation is temporally coupled to epithelial damage severity, with immune cell composition evolving during SPEM progression and resolution [207]. Intrinsic regulatory pathways also modulate ILC2 activity. For instances, androgen signaling suppresses excessive cytokine production, restraining gastric inflammation and limiting metaplastic transformation [208]. Collectively, these findings position ILC2s as dynamic orchestrators integrating epithelial injury signals, immune cell heterogeneity, and temporal remodeling to influence gastric disease trajectories.

#### 4.1.2 Chronic Inflammation Fuels Gastric Adenocarcinoma Mediated by MDSCs

Chronic inflammation triggered by *H. pylori* or gastric tissue injury drives progression from gastritis to gastric adenocarcinoma. Polymorphisms in proinflammatory cytokine genes, such as IL‐1β, TNF‐α, NFKB1, associate with increased cancer risk [209, 210, 211]. Stomach‐specific IL‐1β overexpression in transgenic mice induces spontaneous gastric inflammation and cancer and *Helicobacter felis* infection accelerates this process. IL‐1β mobilizes myeloid‐derived suppressor cells (MDSCs) via the IL‐1RI/NF‐κB pathway [209]. MDSCs exert immunosuppression through PD‐L1 upregulation. Accordingly, anti‐PD‐1 antibody fails to reduce tumor burden in IL1β‐transgenic mice in the presence of MDSCs [212].

NF‐κB1 deficiency in mice leads to spontaneous intestinal‐type gastric adenocarcinoma dependent on microorganism [210]. Deficiency elevates TNF, IL‐6, IL‐22, and IL‐11 expression, driving aberrant STAT1 activation. Genetic depletion of either TNF or STAT1 prevents invasive GC development [106, 210]. These studies establish the critical link between local inflammation and gastric carcinogenesis, identifying potential diagnostic and immunotherapeutic targets.

#### 4.1.3 Neutrophils

Neutrophils exhibit dual pro‐ and antitumor functions in GC. Recent work identified CD44^−^CXCR2^−^ neutrophils as tumor‐specific populations, revealing an essential role for the YAP/TAZ‐CD54 axis [213]. In response to GC cell‐derived GM‐CSF, activated neutrophils suppress effector T‐cell proliferation and IFNγ production, promoting GC progression in mice [214]. GC cell‐derived exosomes deliver HMGB1 to induce N2‐polarized neutrophils, which reciprocally enhance GC cell migration [215]. These findings provide strategic insights for developing neutrophil‐based antitumor therapeutics.

#### 4.1.4 Macrophages

Macrophages critically orchestrate gastric carcinogenesis, progression, and immune escape. Tumor‐associated macrophages (TAMs) promote tumor progression through multiple mechanisms, inducing genetic instability, sustaining cancer stem cells, facilitating metastasis, and suppressing adaptive immunity [216, 217, 218]. TAMs are broadly categorized into M1 (antitumor) and M2 (protumor) polarization states.

Multiple signaling pathways regulate TAM polarization. STING modulation, both knockdown and activation, promotes proinflammatory TAM polarization and induces GC cell apoptosis via the IL‐6R‐JAK‐IL‐24 pathway [219]. Knockdown of Dickkopf‐1 (Dkk1), a secreted antagonist of canonical Wnt signaling [220], promotes M1 polarization while inhibiting M2 polarization [221]. The neutralizing monoclonal antibody DKN‐01 inhibits GC growth by blocking M2 polarization through cGAS‐STING pathway activation [221, 222]. The nuclear envelope proteins SUN1/2 act as mechanoregulators during macrophage M1 polarization [223].

Metabolic reprogramming represents an emerging perspective in TAM polarization [224]. Ubiquitin‐specific protease 14 (USP14) correlates with poor prognosis and immunosuppressive phenotypes [225]. USP14 inhibition blocks M1‐like macrophages polarization [226], while its activation stabilizes SIRT1, driving fatty acid oxidation and immunosuppressive M2 polarization [227]. USP14 inhibition disrupts protumoral macrophage activity and remodels the immune microenvironment [227]. Increased lipid uptake by GC cells upregulates PI3K‐γ, polarizing TAMs toward an M2‐like state with reduced phagocytosis and elevated PD‐L1 expression, blocking antitumor T‐cell responses [228]. Targeting macrophage lipid metabolism thus presents a promising therapeutic strategy [227].

### 4.2 Adaptive Immunity

#### 4.2.1 B Cells and Tertiary Lymphoid Structures in GC

Tumor‐infiltrating B cells correlate with improved overall survival in GC and serve as an independent protective prognostic factor [229]. B‐cell recruitment to the TME is guided by CCL21 and CXCL13 chemokine gradients [230]. B cells restrain tumor progression through three primary mechanisms: (1) antibody‐mediated cytotoxicity via secretion of tumor‐specific antibodies promotes ADCC by NK cells and ADCP by phagocytes; (2) antigen presentation to prime tumor‐specific T cells; and (3) direct killing via granzyme B production [230, 231].

Single‐cell analysis reveals increased antibody‐secreting B cells in diffuse‐type versus intestinal‐type GC [232]. Natural antibodies against sulfated glycosaminoglycans were identified as major functional B‐cell antigens in diffuse‐type gastric carcinoma, which significantly inhibits GC cell growth [233].

Within the TME, B cells aggregate in tertiary lymphoid structures (TLSs), an ectopic lymphoid organizations analogous to secondary lymphoid follicles. TLSs primarily contain follicular dendritic cells surrounded by CD3^+^ T cells and high endothelial venules [234, 235]. B cells and TLSs improve survival and enhance immune checkpoint blockade (ICB) efficacy in multiple cancers [236, 237, 238]. In GC, tumor‐infiltrating B cells and TLS formation correlate with favorable patient prognosis [239, 240].

B cells can also exert protumorigenic effects through immunosuppressive molecules, including IL‐10, IL‐35, TGF‐β, and GABA [230, 241]. IL‐10‐producing regulatory B cells within the CD19^+^CD24^hi^CD27^+^ subset were reported to accumulate in human GC tissues, suppress autologous CD4^+^ T‐cell proliferation and IFNγ production, correlating with shorter overall survival [242].

#### 4.2.2 Prognosis Value and Functional Heterogeneity of T Cells in GC

##### 4.2.2.1 Core Concept

Tumor‐infiltrating T cells exhibit profound functional heterogeneity. The balance between cytotoxic effectors, exhausted cells, and immunosuppressive subsets determines clinical outcomes and immunotherapy response.

T lymphocytes constitute a crucial arm of adaptive antitumor immunity. However, the association between CD8^+^ T‐cell infiltration and clinical outcomes in GC remains inconsistent, which reflects the profound heterogeneity and plasticity of tumor‐infiltrating T cells [240]. Higher intratumoral densities of specific subsets, such as CD103^+^CD8^+^ tissue‐resident memory T cells or CXCR5^+^CD8^+^ T cells, correlate with improved overall survival and greater benefit from adjuvant chemotherapy [243, 244]. Paradoxically, other studies demonstrate that bulk CD8^+^ T‐cell density positively associates with PD‐L1 expression in tumor and stromal regions, predicting worse progression‐free and overall survival [245]. Similarly, elevated CXCL13^+^CD8^+^ T‐cell infiltration correlates with poor clinical outcomes and reduced responsiveness to fluorouracil‐based adjuvant chemotherapy [246]. These seemingly contradictory findings underscore the importance of subset‐specific analysis rather than bulk T‐cell quantification.

Despite infiltration by tumor‐reactive effector T cells, cancer cells employ diverse mechanisms to evade immune surveillance. The immunosuppressive microenvironment, characterized by nutrient deprivation, hypoxia, adenosine accumulation, chronic antigen exposure, and upregulated immune checkpoint ligands, drives T‐cell dysfunction. Exhausted T cells exhibit upregulated coinhibitory receptors such as PD‐1, CTLA‐4, TIM‐3, TIGIT, and LAG‐3. They also show impaired effector cytokine production and profound epigenetic, transcriptional, and metabolic alterations [247, 248]. Notably, while PD‐1 expression increases on tumor‐infiltrating CD8^+^ T cells in GC, PD‐1^+^CD8^+^ T cells retain effector cytokine production comparable to PD‐1^−^ counterparts, suggesting PD‐1 alone may not mark dysfunction in GC [249]. In contrast, TIGIT^+^CD8^+^ T cells exhibit bona fide functional exhaustion with impaired activation, proliferation and glycolysis. CD155, a TIGIT ligand highly expressed in GC tissues and cell lines, promotes this CD8^+^ T‐cell dysfunction [250]. Furthermore, unconventional IL‐17‐producing CD8^+^ T cells (Tc17) accumulate in tumors and predict poor outcomes by promoting MDSC recruitment via tumor‐derived CXCL12 [251].

Dysregulated T‐cell activation in local gastritis promotes gastric hyperplasia and adenocarcinoma. T‐cell‐specific deletion of the tumor suppressor liver kinase B1 (LKB1) leads to excessive production of proinflammatory cytokines and chemokines (IL‐6, IL‐11, CXCL2), enhanced STAT3 activation and infiltration of inflammatory monocytes and neutrophils. This LKB1 deficiency‐driven inflammation promotes gastrointestinal polyposis, which is a cancer predisposition syndrome [252].

Autoimmune gastritis, mediated by self‐reactive CD4^+^ T cells targeting H^+^/K^+^‐ATPase on parietal cells, also drives gastric carcinogenesis. In TCR‐transgenic mice, chronic gastritis features dense CD4^+^ T‐cell infiltration with elevated IFNγ and IL‐17 production, progressing from oxyntic atrophy through mucinous hyperplasia, SPEM, and finally to intraepithelial neoplasia [253].

#### 4.2.3 Treg Cells in the GC Microenvironment

Beyond conventional T‐cell subsets, regulatory T cells (Tregs) constitute a major immunosuppressive component of the GC microenvironment. In *H. felis*/N‐methyl‐N‐nitrosourea (MNU)‐induced GC mouse models [254], Treg depletion attenuates tumor progression, evidenced by reduced tumor area and increased effector T‐cell infiltration [255]. Tumor‐derived TNF‐α induces an effector and memory phenotype (CD45RA^−^CCR7^−^) in tumor‐infiltrating Tregs, enhancing their suppressive capacity [256]. Notably, GCs with RHOA Y42 mutations show elevated Treg infiltration and reduced CD8^+^ T cells [257]. Mutant RHOA activates the PI3K‐AKT‐mTORC pathway to increase FASN‐dependent free fatty acid production and Tregs preferentially uptake and utilize these fatty acids, revealing how tumor metabolic reprogramming establishes immunosuppressive niches.

Targeting Tregs represents a promising GC therapeutic strategy, yet selectively inhibiting tumor‐infiltrating Tregs without disrupting peripheral Treg homeostasis remains challenging. Potential tumor‐infiltrating Treg‐specific targets include CD25, CTLA‐4, GITR, CCR4, CCR8, CXCR3, and PF4 [258, 259, 260]. Recently, we demonstrated that disrupting the p97–Npl4 interaction selectively inhibits tumor‐infiltrating Treg development and enhances antitumor immunity in preclinical models [261], offering a novel strategy to overcome Treg‐mediated immunosuppression in GC.

### 4.3 Other TME Components

#### 4.3.1 Cancer‐Associated Fibroblasts

Cancer‐associated fibroblasts (CAFs) are major stromal constituents in GC and critically influence tumor progression through microenvironmental remodeling [262]. They modulate the cellular niche by secreting of extracellular matrix proteins, growth factors, proteases, cytokines, and chemokines [263], establishing complex signaling networks with cancer cells that facilitate metastatic dissemination [264, 265].

Metabolic crosstalk between CAFs and immune cells shapes the TME. CAFs expressing nicotinamide N‐methyltransferase, together with macrophages expressing nicotinamide phosphoribosyltransferase, regulates nicotinamide/methylnicotinamide ratios to modulate CD8^+^ T‐cell function, which is a metabolic “face‐off” mechanism [266]. Wnt5a signaling dysregulates miRNA expression in gastric CAFs, promoting cancer cell migration [263], while cytonemes mediate Wnt receptor transfer from CAFs to GC cells, enabling Wnt/planar cell polarity pathway responses [262].

Hyaluronan and proteoglycan link protein 1 is the most significantly upregulated gene in GC CAFs, with elevated levels correlating with poor survival [267]. Its expression is induced by GC cells via TGF‐β1/Smad2/3 signaling to promote tumor migration and invasion [267]. CAFs also impair NK cell antitumor function through iron‐dependent mechanisms. They export iron to the TME while upregulating iron regulatory genes such as ferroportin1 and hephaestin, increasing the labile iron pool in NK cells and compromising cytotoxicity [268, 269].

#### 4.3.2 Endothelial Cells

The tumor endothelium forms a critical barrier regulating leukocyte trafficking, nutrient delivery and immune surveillance, representing both a biological prerequisite for cancer progression and a therapeutic vulnerability [270, 271]. Tumor‐associated endothelial cells promote GC growth and metastasis by upregulating Wnt signaling and angiogenic activity [272].

GC mesenchymal stem cells stimulate endothelial cell proliferation, migration and angiogenesis [273, 274] promoting GC cell migration and invasion by inducing Slit2 expression in endothelial cells via AKT signaling [275]. Endothelial protein C receptor enhances GC cell proliferation and migration via PAR1‐mediated ERK1/2 and AKT activation [276, 277].

Vasculogenic mimicry constitutes an endothelial‐independent blood supply system in GC, correlating with poor prognosis and altered immune infiltration [278, 279]. Apatinib, a selective VEGFR‐2 inhibitor, improves survival in advanced GC [280], but resistance remains a challenge. GC‐derived exosomal miR‐214‐3p targets A20 in endothelial cells, suppressing ACSL4‐mediated lipid peroxidation and reducing apatinib efficacy. Inhibiting miR‐214‐3p enhances endothelial sensitivity to the drug [281].

#### 4.3.3 The Extracellular Matrix

The extracellular matrix (ECM) is a noncellular network of fibrous proteins, proteoglycans, cytokines, growth factors, matrix metalloproteinases (MMPs) and hormones that maintains epithelial tissue architecture [282]. Through matrix stiffness, integrin signaling, and cytokine signaling, the ECM regulates differentiation, proliferation, survival, adhesion, and migration of cancer cells.

Gastric carcinogenesis involves extensive ECM dysregulation, characterized by increased collagen deposition, enhanced stiffness, and aberrant integrin expression [283]. Recent studies have provided new insights into the roles of the ECM in GC pathogenesis, immune regulation, metastasis, and therapy resistance. For instance, collagen I inhibits diffuse‐type GC differentiation, augmenting malignant phenotypes [284]. FERMT2 stabilizes SOX2 upregulating FN1 transcription to strengthen cell–matrix interactions and confer anoikis resistance [285]. DDR1 promotes GC progression by stabilizing HIF‐1, facilitating angiogenesis and cytoskeletal reorganization [286]. Post‐translational modifications also modulate tumor–ECM interactions. Succinylation of fibrillin‐1 protects it from MMP‐mediated degradation, activating TGF‐β1 signaling and downstream PI3K/Akt pathway [287].

ECM remodeling by other cellular components influences disease progression. GC cells reprogram CAFs to upregulate HAPLN1 expression, enhancing tumor migration and invasion [267]. The ECM protein EMILIN‐1 maintains lymphatic vessel integrity, with its loss promoting GC development [288]. ECM stiffness emerges as a critical mechanical cue, promoting metastasis and chemoresistance by regulating signaling pathways and facilitating mitochondrial transfer [289, 290]. The interaction between mechanical forces and cellular metabolism has also been documented, whereby tumor cells may sense and respond to ECM rigidity, resulting in enhanced proliferative and metastatic capabilities [291]. Thus, ECM components, MMP activity, and mechanical stiffness are intricately linked to GC initiation, progression, metastasis, and drug resistance, positioning the ECM as a promising therapeutic frontier.

#### 4.3.4 Cytokines

Cytokines enriched in the GC microenvironment, including IL‐1β, IL‐6, IL‐8, IL‐10, IL‐17, TNF, and TGF‐β, play diverse roles in tumorigenesis, progression, metastasis, and therapy resistance [292]. Hosts carrying proinflammatory cytokine gene polymorphisms exhibit higher GC susceptibility following *H. pylori* infection [293, 294]. Immunosuppressive cytokines such as IL‐6, IL‐10, and TGF‐β promote immune evasion and immunotherapy resistance [295, 296, 297].

Recent studies have elucidated cytokine‐mediated crosstalk between GC cells and CAFs. GC cells can promote fibroblast‐to‐CAF conversion via the IL‐17–NFkB axis. These CAFs in turn secrete IL‐8 to fuel tumor malignancy, establishing a positive feedback loop [298]. Inflammatory cytokines such as IL‐1α, IL‐1β, TNF secreted by GC cells induce a senescence‐associated secretory phenotype in CAFs, facilitating peritoneal metastasis [299]. CAF‐derived TGF‐β regulates the lncRNA *TGILR* in GC cells, driving metastasis and EMT [300].

Cytokines and cytokine profiles serve as prognostic biomarkers [299, 301]. Cytokine‐based therapy represents a promising strategy, but pleiotropy and nonspecificity can cause severe adverse effects, necessitating improved targeting approaches [302]. Combining cytokine modulation with other treatments offers an alternative strategy [303].

#### 4.3.5 Metabolites

Metabolic reprogramming sustains GC proliferation, invasion and dissemination, characterized by rewired glucose and amino acid utilization, altered central carbon flux, and elevated nitrogen demand.

##### 4.3.5.1 Glucose Metabolism

Cancer cells employ aerobic glycolysis, namely, Warburg effect, for rapid ATP generation and biosynthetic intermediates, resulting in lactate accumulation [304, 305]. HIF‐1α can activates glycolytic genes and correlates with poor GC prognosis [306]. Insulin signaling [307, 308], PI3K‐Akt‐mTOR pathway [309, 310], and SETD1A‐mediated epigenetic control (previously mentioned H3K4me3 on *HK2/PFKFB3* promoters) [164] enhance glycolysis. Dysregulated glucose metabolism induces YAP hyperactivation, driving GC development [98, 99].

##### 4.3.5.2 Lipids Metabolism

Tumor‐derived lipids polarize TAMs toward M2‐like states [311] and may impair tissue‐resident memory T‐cell survival [312]. Fatty acid–CD36 axis promotes GC metastasis [313]. GC cells exhibit enhanced lipogenesis and upregulated mitochondrial fatty acid β‐oxidation [314, 315], yielding β‐hydroxybutyrate as a predominant byproduct [315]. Adipocyte lipolysis fuels these processes [316], explaining GC's metastatic tropism for adipose‐rich sites and the observation that high‐fat diets promote peritoneal dissemination [317, 318]. Cholesterol metabolism is equally pivotal, with GC patients showing decreased high‐density lipoprotein cholesterol and elevated low‐density lipoprotein cholesterol levels [319, 320].

##### 4.3.5.3 Amino Acid Metabolism

Plasma and tissue analyses reveal GC‐specific amino acid alterations, with glutamine, ornithine, histidine, arginine, and tryptophan decreased [321], while alanine, arginine, glycine, methionine, phenylalanine, valine, and proline increased in GC [322, 323]. Glutamine, the second most critical nutrient after glucose [304, 324], drives GC progression via c‐MYC‐mediated metabolic rewiring [325] and SNAT2 overexpression [326]. Targeting glutamine synthesis may suppress GC growth [327].

### 4.4 Immune Surveillance and Tumor Evasion

Immune escape enables malignant progression by reducing tumor immunogenicity and establishing an immunosuppressive microenvironment [95, 328]. Major mechanisms include PD‐L1 upregulation on tumor cells to blunt T‐cell cytotoxicity [245], altered MHC expression impairing antigen recognition [329], and cytokine‐driven myeloid reprogramming. Tumor‐derived GM‐CSF activates JAK/STAT3 signaling in neutrophils to induce PD‐L1 expression [214]. IL‐11–IL‐33 signaling activates mast cells to produce macrophage chemoattractants such as CSF2, CCL3, IL‐6, promoting TAM recruitment and reinforcing immunosuppression [330].

EBV also promotes immune evasion through several mechanisms. For instance, viral miRNAs, such as EBV‐miR‐BART11 and EBV‐miR‐BART17‐3p, enhance PD‐L1 transcription by targeting FOXP1 and PBRM1 [331]. LMP2A‐activated PI3K/AKT pathway can upregulate tissue factor F3, promoting platelet activation and dampening NK‐cell antitumor activity [332].

## 5 Progression and Metastasis

**FIGURE 4:**
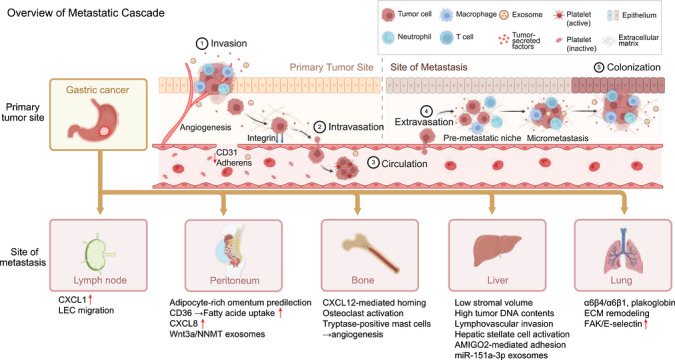
Organotropic metastatic cascade of gastric cancer. This schematic summarizes the major steps of gastric cancer metastasis, from primary tumor invasion and vascular dissemination to distant seeding and colonization. It also highlights representative interactions between tumor cells and the surrounding stromal, immune, vascular, and extracellular matrix compartments during metastatic progression. Common metastatic sites, including lymph nodes, peritoneum, bone, liver, and lung, are shown together with selected features associated with organ‐specific dissemination. Created with BioRender.com.

Metastasis is responsible for most GC‐related deaths, yet the underlying mechanisms remain incompletely understood. This section reviews the current knowledge of GC metastasis, focusing on the organ‐specific and shared metastatic pathways (Figure 4).

### 5.1 Organotropic Metastasis

Despite advances in multidisciplinary therapy and surgery, GC prognosis remains poor due predominantly to metastasis, which accounts for most advanced‐stage deaths [333, 334, 335, 336]. GC metastasis is characterized by high heterogeneity, local tissue invasion, immunomodulation, lymphovascular spread, and hematogenous dissemination [337], with distinct organotropism toward lymph nodes, peritoneum, liver, lung, bone, ovaries, and brain [338].

#### 5.1.1 Lymphatic Metastasis

Lymph node metastasis is common in GC and critically influences prognosis and treatment [339]. Mechanistically, GC cells induce CXCL1 secretion from lymphatic endothelial cells (LECs) via NF‐κB, stimulating FAK‐ERK1/2‐RhoA‐mediated LEC migration and duct formation to facilitate metastasis [340]. Tumor‐derived extracellular vesicles (EVs) further disrupt endothelial barriers by inducing cytoskeletal rearrangement, downregulating adherens junctions, and CD31, and creating transmigration gaps for cancer cells [341].

#### 5.1.2 Peritoneal Metastasis

Peritoneal metastasis is the leading cause of GC recurrence and correlates with poor prognosis [342]. This aggressive process involves tumor cell shedding, migration, adhesion, invasion, and angiogenesis [343], culminating in refractory ascites and bowel obstruction [344, 345]. Key mechanisms include: (1) predilection for adipocyte‐rich sites such as the omentum [346]; (2) hypoxia‐induced CD36 expression promoting fatty acid uptake and metastatic progression [347]; (3) expansion of proangiogenic, antigen‐presentation‐deficient monocyte‐like dendritic cells [348]; (4) FAK phosphorylation‐mediated regulation of Claudin‐1 (inhibited by β‐elemene) [349]; and (5) CXCL8 signaling that enhances proliferation, migration, invasion, epithelial–mesenchymal transition, angiogenesis, and metastasis [343, 350, 351].

#### 5.1.3 Liver Metastasis

##### 5.1.3.1 Core Concept

Liver metastasis, the most frequent distant metastatic site in GC, is driven by specific adhesion molecules, hepatic stellate cell activation, and premetastatic niche formation.

As the most frequent distant metastatic site [352], liver metastasis associates with low stromal volume and high tumor DNA content [353]. COX‐2 overexpression represents an additional risk factor [354]. Critical mechanisms include: (1) Adhesion and colonization: AMIGO2‐mediated tumor cell adhesion to hepatic endothelium facilitates initial seeding [355, 356]; (2) Premetastatic niche formation: Lipopolysaccharide‐binding protein activates TLR4/NF‐κB signaling in hepatic cells, inducing TGF‐β1 secretion that activates hepatic stellate cells to form a fibrotic premetastatic niche [357]; (3) Immunomodulation: MAPK4 knockdown in tumor cells promotes macrophage migration inhibitory factor secretion, polarizing TAMs to an M2‐like state that accelerate liver metastasis [358]; (4) Lymphovascular involvement: Vascular invasion serves as an independent predictor of synchronous liver metastasis [359].

#### 5.1.4 Lung Metastasis

Lung metastasis is the third most common metastatic site in GC patients. Unlike liver and peritoneal metastases, which often occurs independently, lung metastasis frequently coexists with liver metastasis [352]. Although intravenous injection models are commonly used to study GC lung metastasis [360, 361, 362], the underlying mechanisms driving lung tropism remain poorly understood. Potential mechanisms include: (1) desmosomal markers (e.g., plakoglobin) facilitating lymphatic invasion and pulmonary homing [363]; (2) exosomal integrins α6β4/α6β1 establishing lung‐specific premetastatic niches [364]; (3) unique lung physiological features including dense capillaries and intact basement membranes [365]; (4) hematopoietic progenitor cells promoting fibronectin binding and MMP9 secretion to remodel extracellular matrix [366, 367, 368], and (5) FAK/E‐selectin activation enhancing endothelial permeability for circulating tumor cell extravasation [369].

#### 5.1.5 Bone Metastasis

Bone metastasis occurs in approximately 12% of GC cases [352, 370] and confers a median survival of only 6.5 months [370, 371, 372]. Proposed mechanisms include: (1) CXCL12‐mediated homing to bone niches [373, 374]; (2) osteoclast‐driven release of trophic factors (growth factors, calcium, cytokines) that support tumor growth [365]; (3) tumor cell expression of bone turnover factors, which in GC are often RANKL‐independent [375]; and (4) tryptase‐positive mast cells stimulating angiogenesis at both primary and metastatic sites [376].

### 5.2 Mechanisms of GC Metastasis

Most GC patients present with advanced metastatic disease precluding curative resection, and advanced GC carries a dismal prognosis with < 15% 5‐year survival [94]. Metastasis is a multistep, microenvironment‐dependent cascade involving EMT‐driven invasion, ECM remodeling, immune evasion, and organ‐specific niche conditioning [377].

#### 5.2.1 Angiogenesis

Angiogenesis helps sustain metastatic growth by supplying nutrients, which encompasses both endothelial vessel formation and vasculogenic mimicry [94]. Several angiogenic factors and cytokines secreted from cancer cells drive this process, including VEGF [378], IL‐8 [379], FGF‐2 [380], and PD‐ECGF [381]. Stromal components such as CAF or TAM‐derived COX‐2 were also involved [382]. In addition, CAF‐derived AGR2 activates hypoxia signaling and promotes neovascularization [383]. Following niche establishment, GC cells penetrate the ECM and intravasate into the vasculature to enable distal dissemination.

#### 5.2.2 Extracellular Vesicles

EVs critically orchestrate GC metastasis through organ‐specific niche modulation. GC‐derived Wnt3a‐positive EVs facilitate peritoneal mesothelial cell infiltration and subserosal invasion [384]. Elevated exosomal NNMT correlates with peritoneal metastasis [385] and activates TGF‐β/ Smad2 signaling in mesothelial cells [386]. During liver metastasis, GC‐derived EV cargo such as miR‐151a‐3p activates TGF‐β/Smad signaling in Kupffer cells, promoting a stem cell‐permissive niche [386].

## 6 Therapeutic Advancements

**FIGURE 5:**
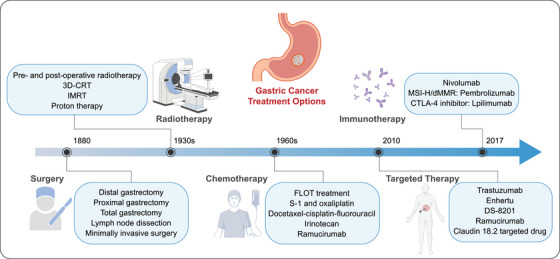
The development of therapies for GC. The treatment landscape of gastric cancer has expanded from a surgery‐centered paradigm to a multimodal framework that integrates radiotherapy, systemic chemotherapy, molecularly targeted agents, and immunotherapy. This therapeutic evolution reflects a broader shift from anatomical tumor control toward biologically informed and increasingly personalized intervention. Together, these advances have progressively diversified clinical options across disease stages and created new opportunities for stratified treatment in gastric cancer.

While surgical resection remains foundational, treatment strategies should integrate patient‐specific factors, including performance status, tumor pathology, invasion extent, and developmental trajectory, with planned application of available modalities to achieve radical tumor control, improved prognosis and enhanced quality of life (Figure 5).

### 6.1 Endoscopic Resection

Advances in early GC detection and therapeutic endoscopy have established endoscopic resection as standard practice [387]. Following histological confirmation via endoscopic biopsy, GC staging integrates computed tomography and endoscopic ultrasound. Endoscopic mucosal resection (EMR) and endoscopic submucosal dissection (ESD) represent the primary techniques for treating gastric epithelial neoplasia, including early carcinoma [388], with ESD as the gold standard for early GC. Clinical indications for ESD are stratified based on lymph node metastasis risk, primarily governed by histology, depth of invasion, and ulceration [389, 390]. Compared to EMR, ESD achieves superior en bloc, complete, and curative resection rates with reduced local recurrence. This minimally invasive approach demonstrates low morbidity while preserving organ function and quality of life.

### 6.2 Surgery and Lymphadenectomy

Surgical management of GC primarily involves subtotal or total gastrectomy. This approach is indicated for clinically staged T1 with nodal involvement (N+), T2–T4a tumors (any N status), and cases without distant metastases. Surgical resection aims to achieve local radical resection with negative margins. Given GC's poorly differentiated nature and diffuse growth pattern, wide excision is essential. Key principles include tumor‐free resection margins, regional lymph node clearance, and perioperative/adjuvant chemotherapy integration [391].

The primary surgical modalities are distal gastrectomy and total gastrectomy. Distal gastrectomy, involving resection of the distal two‐thirds of the stomach followed by anastomosis of the proximal stomach to the small intestine, is indicated primarily for tumors not involving the esophagogastric junction. Total gastrectomy entails anastomosing the esophagus directly to the small intestine. Both procedures require pyloric resection, which allows rapid bolus transit into the small intestine and may lead to long‐term complications such as dumping syndrome and weight loss [391, 392]. Pylorus‐preserving alternatives, including pylorus‐preserving gastrectomy or proximal gastrectomy with double‐tract reconstruction, show promise for mitigating dumping symptoms and weight loss [94, 393].

Lymphadenectomy extent is determined by metastatic involvement and has been extensively debated. D1 dissection removes perigastric and left gastric artery lymph nodes. D2 dissection encompasses all D1 nodes plus those along the common hepatic, proper hepatic, and splenic arteries excluding splenic hilar and celiac axis nodes. D3 dissection extends further to include all D2 stations, supplemented by para‐aortic and hepatoduodenal lymph nodes [394].

### 6.3 Perioperative Treatment of GC

#### 6.3.1 Neoadjuvant and Perioperative Chemotherapy

Given the pronounced epidemiological, clinicopathological, and biological differences between Eastern and Western GC populations, perioperative treatment strategies have evolved along partially distinct trajectories. In Western cohorts, robust evidence demonstrates that perioperative therapy confers clear advantages over surgery alone [395, 396]. In contrast, data from East Asia indicate that neoadjuvant chemotherapy administered prior to radical gastrectomy enhances pathological response and R0 resection rates while maintaining acceptable safety profiles [397, 398]. The optimal integration of perioperative chemoradiotherapy (CRT) versus postoperative chemotherapy following D2 gastrectomy remains under active investigation [399].

Historically, perioperative chemotherapy has been dominated by fluoropyrimidine‐ and platinum‐based regimens. The landmark MAGIC trial established the survival benefit of perioperative ECF (epirubicin, cisplatin, fluorouracil) compared with surgery alone in operable gastroesophageal adenocarcinoma [396]. Subsequent trials refined regimen selection. The Chinese RESOLVE Phase III study evaluated adjuvant XELOX, adjuvant SOX and perioperative SOX (neoadjuvant plus adjuvant cycles) following D2 gastrectomy, supporting perioperative approaches in locally advanced GC [400]. More recently, the FLOT4‐AIO Phase II/III trial demonstrated superior overall survival with perioperative FLOT compared with ECF/ECX, establishing FLOT as a preferred standard in fit patients with resectable disease [401]. Current neoadjuvant options therefore include XELOX [402], FOLFOX [403], SP (cisplatin/S‐1) [404], and SOX [405], tailored according to regional practice and patient fitness.

Emerging evidence underscores the necessity of patient stratification in neoadjuvant decision‐making. Tumor stage, performance status, and comorbidities remain foundational clinical determinants. However, molecular and immunological features, including HER2 status, microsatellite instability, EBV positivity, and baseline immune infiltration, may predict differential sensitivity to cytotoxic and immune‐based neoadjuvant strategies [406]. Such stratification frameworks aim to identify patients most likely to benefit from intensified perioperative therapy while sparing low‐risk or frail individuals from overtreatment.

Combination strategies integrating immunotherapy into the neoadjuvant setting are gaining momentum. Early‐phase trials suggest that combining immune checkpoint inhibitors with chemotherapy can enhance pathological response rates and potentially induce durable antitumor immunity by exploiting chemotherapy‐induced immunogenic cell death [407]. These approaches appear particularly promising in immunogenic GC subtypes, including MSI‐high and EBV‐positive tumors [408].

Collectively, perioperative and neoadjuvant therapies in GC not only improve the likelihood of curative resection and eradicate micrometastatic disease but also provide a unique in vivo platform to assess treatment response and refine risk‐adapted strategies [396, 409, 410, 411]. Ongoing trials incorporating molecular stratification and combination regimens are expected to further redefine perioperative standards.

#### 6.3.2 Postoperative Adjuvant Chemotherapy

Postoperative adjuvant chemotherapy for GC primarily utilizes fluoropyrimidine‐based regimens (S‐1 monotherapy) or combination therapies. This approach represents standard care for Asian patients. Landmark studies from Japan and Korea demonstrate improved overall survival with adjuvant chemotherapy when combined with D2 lymphadenectomy [412, 413]. Conversely, trials in non‐Asian populations show no survival benefit, a discrepancy attributed to inconsistent surgical standardization where D2 dissection was not systematically performed. A comprehensive individual patient‐level meta‐analysis confirms a moderate absolute survival benefit of 6% for 5‐fluorouracil‐based adjuvant chemotherapy versus surgery alone [414]. Notably, poorer tolerability of adjuvant versus neoadjuvant/perioperative chemotherapy favors the latter approach outside East Asia, ensuring systemic treatment delivery even when postoperative therapy cannot be completed [415].

#### 6.3.3 Adjuvant Chemoradiotherapy: Current Evidence and Refined Indications

The role of adjuvant radiotherapy remains uncertain. Current guidelines do not recommend adding postoperative radiotherapy to perioperative or adjuvant chemotherapy. Randomized trials confirm that radiotherapy after quality‐assured gastrectomy provides no overall survival benefit over D1/D2 lymphadenectomy alone [414, 416]. While the ARTIST trial suggested potential benefit for node‐positive disease with radiotherapy–chemotherapy combinations, ARTIST2 failed to validate this [416, 417].

The INT 0116 trial reported a 9‐month median OS improvement with adjuvant CRT versus observation postcurative surgery [94]. However, only 10% of participants underwent D2 dissection, suggesting this benefit may compensate for inadequate surgery rather than augment optimal resection. Subsequent trials comparing adjuvant chemotherapy to CRT yielded conflicting results [418, 419, 420]. Per NCCN guidelines [421], adjuvant CRT is indicated for R1/R2 resections, with category 1 recommendation for pT3–pT4 or pN+ disease with sub‐D2 dissection. National Cancer Database trends show declining postoperative CRT use alongside rising perioperative chemotherapy adoption [422], reflecting improved tolerance of preoperative regimens, concerns about postoperative toxicity, and recognition of D2 lymphadenectomy's importance.

### 6.4 Therapy for Patients With Advanced GC

Surgical intervention is contraindicated for GC in the following scenarios: (1) Tumor‐related factors: Extensive local invasion where the primary tumor cannot be separated from adjacent structures or encases major vascular structures; regional lymph nodes that are fixed and matted, or metastatic lymph nodes beyond the surgical field; distant metastasis or peritoneal implants including positive peritoneal cytology. (2) Patient‐related factors: Poor overall health status, malnutrition, severe hypoalbuminemia, anemia, significant comorbidities, or patient refusal.

#### 6.4.1 Chemotherapy

Chemotherapy improves survival in patients with locally advanced unresectable or metastatic GC [423]. Compared to patients receiving supportive care alone showing median OS of 3–4 months, patients receiving combination chemotherapy achieve a median OS of approximately 1 year, with Asian patients tending toward slightly longer survival [424]. Therefore, chemotherapy should be offered to patients with good performance status and adequate organ function [425, 426].

Effective chemotherapeutic agents include fluoropyrimidines (5‐fluorouracil, capecitabine, S‐1, and trifluridine–tipiracil), platinum agents, taxanes, and irinotecan. For the initial treatment of metastatic GC, a platinum–fluoropyrimidine doublet is the recommended first‐line regimen [425, 426]. Cisplatin and oxaliplatin demonstrate comparable efficacy but different toxicity profiles. Cisplatin is associated with thromboembolic events and renal insufficiency, while oxaliplatin is associated with neuropathy and diarrhea. A recent randomized Phase III trial found that adding docetaxel to cisplatin and S‐1 did not improve survival compared to doublet therapy [427].

#### 6.4.2 Chemoradiotherapy

For inoperable patients with advanced GC and adequate performance status, concurrent CRT is recommended if the tumor is localized. CRT is superior to chemotherapy or radiotherapy alone in achieving tumor downstaging and pathological response [428, 429]. For patients with extensive tumor invasion or lymph node metastasis, the large irradiation fields required may lead to treatment intolerance. Chemotherapy or radiotherapy alone can be considered in these cases [429]. Patients demonstrating good response should be referred to an MDT to assess the possibility of surgical resection.

For patients presenting with severe complications such as gastrointestinal obstruction, hemorrhage, or obstructive jaundice, initial management should focus on symptom palliation. This may include gastrostomy, stent placement, gastrointestinal bypass surgery, local palliative radiotherapy, proton pump inhibitors, or analgesia, tailored to the clinical situation [430]. Chemotherapy can be considered once the patient's general condition improves. If performance status does not improve, best supportive care remains appropriate.

#### 6.4.3 Precision Medicine: Biomarker‐Guided Targeted Therapy and Immunotherapy

The past decade has witnessed a paradigm shift from empirical chemotherapy to biomarker‐guided precision medicine in advanced GC, driven by the identification of actionable molecular targets and the success of ICB.

**HER2‐targeted therapy** remains the archetype of precision oncology in GC. Approximately 17%–20% of GC patients exhibit *HER2* (*ERBB2*) gene amplification and HER2 protein overexpression, a feature more common in intestinal‐type tumors of the proximal stomach or gastroesophageal junction [431]. The landmark TOGA trial established that adding trastuzumab to cisplatin–fluoropyrimidine chemotherapy significantly improves overall survival compared to chemotherapy alone in HER2‐positive advanced GC [432]. Subsequent studies have explored combination strategies, with the Phase III trial of pertuzumab + trastuzumab + chemotherapy in HER2‐positive advanced GC, demonstrating a numerically higher ORR (57.0% vs. 48.6%) and a modest OS improvement (HR 0.85) that did not reach statistical significance [433].

**CLDN18.2** has emerged as a highly promising target due to its tumor‐specific expression pattern [434]. The monoclonal antibody zolbetuximab demonstrated clinical benefit in Phase III trials, improving outcomes for patients with CLDN18.2‐positive, HER2‐negative advanced GC when combined with chemotherapy [435]. These successes position CLDN18.2 as the second validated therapeutic target in GC after HER2.

**FGFR2** amplification occurs in approximately 9% of GC cases, with significant mutual exclusivity with HER2 amplification [436, 437]. Bemarituzumab, a humanized afucosylated monoclonal antibody against FGFR2b, has shown promising efficacy in FGFR2b‐overexpressing advanced GC and is currently under Phase III evaluation (FORTITUDE‐101) [438, 439].

**ICB** has transformed the treatment landscape for chemotherapy–refractory GC. Nivolumab demonstrated survival benefit versus placebo in the Phase III ATTRACTION‐2 trial involving unselected Asian advanced GC patients [440]. The pivotal Phase III CheckMate‐649 trial established nivolumab + chemotherapy as first‐line standard for metastatic or HER2‐negative GC with PD‐L1 CPS ≥ 5, demonstrating improved OS (14.4 vs. 11.1 months) and PFS [94, 441, 442]. Beyond PD‐1 inhibition, dual checkpoint blockade (nivolumab + ipilimumab) and bispecific antibodies such as cadonilimab targeting PD‐1 and CTLA‐4 are under active investigation, aiming to overcome resistance and enhance efficacy [443, 444].

**Cell cycle dysregulation** offers additional therapeutic opportunities. The CDK4/6 inhibitor palbociclib demonstrates efficacy in GC models with CDKN2A mutations or p16 methylation [445, 446]. However, monotherapy efficacy is limited by intrinsic resistance mechanisms, including TP53 mutations, MDM2/MDM4 overexpression, Hippo pathway inactivation, and Cyclin E‐CDK2 activation [447, 448]. Combinatorial strategies pairing CDK4/6 inhibitors with complementary targeted agents are being actively explored [449].

**Beyond established targets** Chimeric antigen receptor (CAR) T‐cell therapy, engineered to express synthetic receptors targeting tumor‐associated antigens or tumor‐specific antigens, has emerged as a promising approach for solid tumors [450]. In GC, promising targets include HER2, CLDN18.2, mesothelin, EpCAM, and NKG2D [451, 452, 453, 454, 455]. However, CAR‐T efficacy in solid tumors remains limited by T‐cell instability, exhaustion within the suppressive TME and poor tumor infiltration [450, 456]. Mechanistic insights, such as the role of chronic antigen exposure in driving NK‐like dysfunction [457] and the ability of c‐Jun overexpression to confer exhaustion resistance [458], are informing next‐generation strategies to overcome these barriers.

### 6.5 Preclinical Animal Studies of Targeted Therapies in GC

Preclinical targeted strategies for GC increasingly extend beyond established targets to address tumor heterogeneity, therapeutic resistance and the TME. Emerging approaches include novel surface antigens such as CD44v6 and CDH17, next‐generation bispecific antibody and innovative interventions targeting FGFR axis. Complementary TME‐directed strategies such as ECM remodeling, stromal FAP targeting, TGFβR1 inhibition and CD47–SIRPα blockade further enhance antitumor efficacy. Collectively, these integrated tumor–microenvironment targeting approaches represent promising avenues to improve GC outcomes (Table 1).

**TABLE 1:** Preclinical animal studies of targeted therapies in GC.

Target strategy	Agent	Model	Key outcome	References
CD44v6	C44Mab‐9‐mG2a	NUGC‐4 xenograft	Significant tumor growth inhibition	[459]
CDH17	BSI‐721	SNU‐5 xenograft (CDH17‐high)	Significant tumor regression/tumor growth inhibition	[460]
	TAVO307	GI cancer xenograft (including GC)	Robust antitumor activity in CDH17‐expressing models	[461]
HER3 × MET	BCG022	Gastric cancer CDX	Strong tumor growth inhibition; enhanced internalization via dual binding	[462]
PTK7 × TROP2	BCG033	Gastric cancer PDX	Demonstrated broader spectrum/greater potency in in vivo tumor suppression	[463]
FGFR2	LC‐SF‐14	Gastric cancer xenograft	Induced significant tumor regression in vivo	[464]
	N5	Gastric cancer xenograft	Selective degradation of FGFR2 protein led to sustained tumor growth inhibition in vivo	[465]
ECM remodeling	LOX	Gastric cancer xenograft	Reduced collagen crosslinking and suppressed tumor progression	[466]
TGFβR1 inhibition	Galunisertib (LY2157299) ± 5‐FU/paclitaxel	Gastric cancer xenograft	Inhibition of pSmad signaling/EMT and suppression of tumor growth	[467]
CD47–SIRPα blockade	Anti‐CD47 + chemotherapy	Gastric cancer xenograft	Superior tumor growth inhibition	[468]

### 6.6 Ongoing Clinical Trials for Targeted Therapies

Targeted therapies designed to inhibit molecular drivers of GC progression have emerged to improve outcomes. Clinically validated targets include HER2, Claudin 18.2, PARP, FGFR, with corresponding agents currently undergoing clinical evaluation [121, 469, 470, 471, 472, 473]. Immunotherapy advances feature checkpoint inhibitors targeting PD‐1/PD‐L1, CTLA‐4, and TIGIT [474, 475, 476, 477, 478]. Dual‐targeting agents represent a promising frontier. For example, CDX‐585 targeting PD‐1 and ILT4 activates both T cells and myeloid cells, which is currently in Phase I trials for advanced solids (NCT05788484) [479]. These innovative strategies aim to overcome resistance mechanisms and improve outcomes in GC (Table 2).

**TABLE 2:** Ongoing clinical trials with target agents in GC patients.

Target strategy	NCT number	Acronym	Phase	Interventions
HER2	NCT03615326		III	Trastuzumab
	NCT05152147	HERIZON‐GEA‐01	III	Zanidatamab, Trastuzumab
	NCT04249739		II	Trastuzumab
	NCT05190445		II	Cinrebafusp alfa (PRS‐343)
	NCT01191697		II	Trastuzumab
	NCT04513223		I	SHR‐A1811
	NCT04908813		II	HLX22, Trastuzumab
	NCT05586061	RCTS	II	Disitamab vedotin
	NCT04511871		I	CCT303‐406
	NCT04661150		II	Trastuzumab
	NCT04704934		III	Trastuzumab deruxtecan
	NCT04430738		II	Tucatinib, Trastuzumab
	NCT05671822		II	SHR‐A1811
	NCT04492488		I+II	MRG002
	NCT04660929		I	CT‐0508
	NCT04639219	DPT01	II	Trastuzumab deruxtecan
	NCT05382364		I	Tucatinib
	NCT03929666		II	ZW25 (Zanidatamab)
	NCT05504720		II	Trastuzumab
Claudin 18.2	NCT04404595		I + II	CT041
	NCT03505320	ILUSTRO	II	Zolbetuximab
	NCT03653507	GLOW	III	Zolbetuximab
	NCT03504397	Spotlight	III	Zolbetuximab
PARP	NCT02734004	MEDIOLA	I + II	Olaparib
	NCT03008278		I + II	Olaparib
FGFR	NCT04595747		II	Rogaratinib
	NCT05052801	FORTITUDE‐101	III	Bemarituzumab
Antiangiogenesis	NCT04879368	INTEGRATEIIb	III	Regorafenib
	NCT00569127		III	Bevacizumab
	NCT03008278		I + II	Ramucirumab
Immunotherapy	NCT04166721		I + II	Atezolizumab
	NCT04062656	IMAGINE	II	Nivolumab, Relatlimab
	NCT03443856	VESTIGE	II	Nivolumab, Ipilimumab
	NCT06940921		I + II	Cadonilimab
	NCT03776487		II	Ipilimumab, Nivolumab
	NCT05941481	RARE	II	Tislelizumab
	NCT05836584		II	Atezolizumab
	NCT04989387		I	INCA00186, Retifanlimab
	NCT03281369		I + II	Atezolizumab, Tiragolumab
	NCT05788484		I	CDX‐585
	NCT04802876	ACROPOLI	II	Spartalizumab, Tislelizumab
	NCT06739161		II	Sintilimab
	NCT04006262	NEONIPIGA	II	Nivolumab, Ipilimumab
	NCT05572684		I + II	NC410, Pembrolizumab

*Source*: https://clinicaltrials.gov/.

## 7 Future Directions: Integrating Multidimensional Perspectives for the Next Decade

The intricate dissection of GC's biology, spanning cellular origins, immunosuppressive networks, metabolic reprogramming, neural integration, and microbial crosstalk, reveals both the disease's daunting complexity and unprecedented therapeutic opportunities. Future breakthroughs hinge on four strategic imperatives.

### 7.1 Decoding the Spatiotemporal Architecture of the GC Ecosystem

The advent of spatially resolved multiomics, including spatial transcriptomics, proteomics, metabolomics, and advanced live imaging now enables high‐resolution mapping of cellular interactions within the TME [480, 481]. Integrating these approaches with longitudinal single‐cell profiling will decode the dynamic evolution of tumor–stroma–immune–neural networks during disease progression and therapy response. A critical frontier is the rigorous elucidation of the neural–tumor–immune axis, defining how autonomic/sensory nerves and Schwann cells mechanistically influence perineural invasion, angiogenesis, and immunosuppression via neurotransmitters, neurotrophins, and direct cellular crosstalk [8, 9, 482, 483, 484]. This will reveal druggable neural signaling nodes such as neurotransmitter receptors or neurotrophin pathways, and establish GC as a model for “cancer neuroscience” research.

### 7.2 Exploiting Novel Target Classes Beyond the Cancer Cell

Robust functional validation of cell surface molecules, including both proteins and, increasingly, surface RNAs [485, 486, 487], as actionable immune checkpoints is essential to drive development of blocking antibodies or aptamers. Targeting metabolic vulnerabilities demands integrated strategies that disrupt immunosuppressive pathways, including adenosine signaling, lactate shuttling, and tryptophan metabolism, within specific niche contexts, moving beyond single‐enzyme inhibition [117, 488, 489]. Similarly, modulating neural activity via repurposed neuroactive drugs or novel agents targeting receptors like β‐adrenergic or Trk represents an underexplored therapeutic avenue [200, 202, 490, 491]. The microbiome, namely, bacterial, fungal, and viral, offers additional opportunities for therapeutic modulation, either by eliminating pathogenic species or by harnessing microbial metabolites to enhance immunotherapy efficacy [492, 493, 494, 495, 496, 497].

### 7.3 Implementing Intelligent, Biomarker‐Driven Combination Strategies

Overcoming the adaptive resilience of the TME necessitates rational combinatorial approaches that simultaneously address: (a) multiple immunosuppressive cell types, (b) tumor‐intrinsic drivers and microenvironmental modifiers, (c) classical and novel immune checkpoints alongside metabolic or neural inhibitors, and (d) systemic contributors like premetastatic niches. Success depends on biomarker‐driven precision medicine, integrating deep molecular profiling of tumors and TME components, including neural signatures, dominant metabolic pathways, and specific target expression, with dynamic liquid biopsy monitoring such as ctDNA, exosomes, and circulating tumor cells [498]. This integration will guide adaptive selection of combinatorial regimens.

### 7.4 Harnessing Artificial Intelligence as a Translational Accelerator

The advancement of artificial intelligence (AI) has demonstrated transformative potential across the GC care continuum [499]. Beyond endoscopic image analysis for early detection, AI applications are expanding to: (a) predicting treatment response by integrating multimodal data including imaging, genomics, and pathology [500, 501]; (b) accelerating drug discovery by identifying novel targets and predicting drug sensitivity [502]; (c) optimizing clinical trial design through patient stratification and digital twin simulations [503, 504]; and (d) personalizing combination strategies by decoding complex interaction networks from multiomic data [503, 505]. It will be essential for clinical integration to overcome current limitations, including the “black‐box” nature of many models, dependence on training data quality and need for prospective validation [506, 507].

Embracing this holistic strategy, leveraging technological innovation to dissect dynamic interactions, validating neural and noncanonical targets and implementing smart biomarker‐stratified combinations, offers the greatest promise for transforming outcomes in this recalcitrant malignancy. The future of GC therapy lies in the seamless convergence of deep biological insight, interdisciplinary collaboration and AI‐powered computational intelligence, transforming the integrated TME from an insurmountable barrier into a target‐rich therapeutic landscape.

## Author Contributions

Ruixian Yu, Miao Zhang, Chunxiao Zhu, and Yan Meng collected information and wrote most parts of the review. Weihong Zhang and Junping Bai helped to write the adaptive immunity section. Hui Zhang helped to write the GC classification section. Zhifa Cao, Yang Tang, Meihang Du, Zhangting Zhao, and Yi Han helped to write the section of signaling transduction in GC development. Wei Kang and Ka Fai To provided valuable suggestions about the outline of review structure. Ruixian Yu, Miao Zhang, Shi Jiao, Liwei An, and Zhaocai Zhou designed the framework of the review. Shi Jiao, Liwei An, and Zhaocai Zhou supervised, wrote, and revised the review. All the authors have read and approved the final manuscript.

## Funding

This work was supported by the Noncommunicable Chronic Diseases‐National Science and Technology Major Project (2024ZD0533203, 2025ZD0545200), the Innovative Drug Research and Development‐National Science and Technology Major Project (2025ZD1800300), the National Natural Science Foundation of China Grants (82361168638, 82372613, 82373251, U25A20113, 82573553, 32501140), Fund of Fudan University and Cao'ejiang Basic Research (24FCB05), and Shanghai Eastern Talent Program (BJKJ2025031).

## Ethics Statement

The authors have nothing to report.

## Conflicts of Interest

The authors declare no conflicts of interest.

## Data Availability

The authors have nothing to report.
